# Dissolved oxygen concentrations influence microbial community structure and key players in an oxygen minimum zone

**DOI:** 10.1128/msphere.00751-25

**Published:** 2026-07-15

**Authors:** Kaitlin R. Dombroski, Lauren G. Campbell, Robert M. Morris, Olivia U. Mason

**Affiliations:** 1 Department of Earth, Ocean and Atmospheric Science, Florida State University 7823, Tallahassee, Florida, USA; 2 School of Oceanography, University of Washington 189579, Seattle, Washington, USA; Virginia Tech, Blacksburg, Virginia, USA

**Keywords:** nBUS, OMZ, EBUE, SUP05, *Thioglobaceae*, omics, 16S rRNA, Benguela, Namibia, dissolved oxygen, incomplete denitrification, N_2_O, alpha diversity

## Abstract

Oxygen minimum zones (OMZs) are expanding in the global ocean. Declining oxygen can alter microbial processes that regulate carbon, nitrogen, and sulfur cycling, including pathways with the potential to produce nitrous oxide (N₂O), a potent greenhouse gas. Yet, we lack a full understanding of how microbial communities respond to declining oxygen in some globally important OMZs. This is particularly true for the understudied northern Benguela Upwelling System (nBUS), an important source of marine N₂O. Here, we analyzed the influence of oxygen concentrations on microbial communities in the nBUS OMZ using 16S rRNA gene sequence data. Microbial diversity did not decline linearly with oxygen and was highest under dysoxic (0.2–2.0 mL/L) conditions and lowest under suboxic (0.0–0.2 mL/L) conditions. This pattern was primarily due to changes in community evenness, which decreased in suboxic samples as the relative abundance of *Thioglobaceae* (SUP05) increased. *Thioglobaceae* relative abundance was significantly inversely correlated with oxygen, and *Thioglobaceae* were abundant in the nBUS OMZ. Analysis of 216 publicly available medium- to high-quality *Thioglobaceae* genomes from cultured and uncultured microbes sampled in the nBUS and other OMZs revealed the metabolic potential of this clade for carbon fixation and consumption, sulfur oxidation, and denitrification. Importantly, few *Thioglobaceae* possess the genetic potential to carry out complete denitrification as most lack the gene that converts N₂O to dinitrogen gas. As OMZs expand in size and severity, decreasing microbial diversity and a concomitant increase in the relative abundance of *Thioglobaceae* could contribute to enhanced N₂O production through incomplete denitrification.

**IMPORTANCE:**

Marine microbes mediate biogeochemical cycles, which are influenced by oxygen concentrations, including microbial pathways that can lead to nitrous oxide (N₂O) production. The oxygen minimum zone (OMZ) in the northern Benguela Upwelling System (nBUS) is a biogeochemical hotspot and an important source of nitrous oxide. Yet, microbial community responses remain poorly characterized in this understudied OMZ. Analysis of microbial communities in this OMZ, combined with comparative genomic analysis of *Thioglobaceae* from the global ocean, revealed that members of this group have the capacity to fix carbon, potentially supporting growth through sulfur oxidation coupled to denitrification. However, few *Thioglobaceae* have the genetic potential to carry out complete denitrification, with the final nitrous oxide reduction step often missing. Incomplete denitrification is an important source of N₂O in OMZs. Collectively, this study linked oxygen-associated community shifts in this OMZ to incomplete denitrification in a microbial group that increased in relative abundance as oxygen declined.

## INTRODUCTION

Marine oxygen minimum zones (OMZs) are regions where dissolved oxygen (DO) is less than 62 μM/kg or 2 mL/L (1–4). These regions are expanding due to rising ocean temperatures, which decrease oxygen solubility and reduce water column ventilation (2, 5–7). Although low in DO, OMZs are enriched in the by-products of microaerophilic and anaerobic microbial metabolisms, including carbon dioxide, methane, nitrous oxide (N₂O), and hydrogen sulfide (2, 8, 9). For example, as DO declines, microbial production of marine nitrous oxide (N₂O), a potent greenhouse gas, increases, with OMZs accounting for approximately 60% of marine N₂O emissions (10–12), the majority of which results from microbial processes and incomplete denitrification in particular (13).

Declining oxygen concentrations shape community structure and can lead to shifts in both macro- and microfauna (14). Specifically, microbial community composition changes with low DO levels, particularly under dysoxic conditions (15, 16). For example, bacterial diversity and evenness decreased with declining DO in eastern tropical Pacific OMZs (17, 18). In contrast, bacterial richness increased with declining DO in the Hood Canal estuary and eastern tropical North Pacific OMZ (16, 17). Thus, microbial diversity responses to declining oxygen can vary among systems and diversity metrics. However, the current understanding of microbial diversity patterns in relation to oxygen comes largely from better-studied systems (2, 19), and it remains unclear how these patterns apply to understudied OMZs such as the northern Benguela Upwelling System (nBUS).

Consistent with changes in diversity metrics in relation to DO, several taxonomic surveys have identified consistent trends in microbial community composition and changes in abundance along DO gradients, from the anoxic core to the boundaries of OMZs (17). For example, Pseudomonadota, Thermoproteota, Nitrospinota, SAR324, and Nanobdellota abundances can increase in OMZs (17, 20–25). Among these microbial groups, Pseudomonadota, particularly sulfur-oxidizing members of the family *Thioglobaceae* (26–28), historically referred to as SUP05 but now classified as a genus within the *Thioglobaceae,* dominate OMZs, including the nBUS (2). In fact, detoxification of sulfidic waters in the nBUS was partially attributed to abundant *Thioglobaceae* (26).

Chemoautotrophic *Thioglobaceae* use energy gained through the oxidation of reduced sulfur compounds, under oxic or low-to-no-oxygen conditions, to fix inorganic carbon and are therefore important players in marine carbon and sulfur cycles. In the absence of oxygen, such as in OMZs, members of this group use nitrate as a terminal electron acceptor (29). For example, a recent study found that chemoautotrophic *Candidatus* Thioglobus autotrophicus strain EF1 simultaneously respired oxygen and nitrate when DO concentrations were low (<5 µM) and while nitrate was available (30). This led to higher growth rates, enhanced carbon fixation, and elevated gene expression (30). Importantly, previous experiments with *Ca*. T. autotrophicus revealed that N₂O was produced as a result of incomplete denitrification (31). The genome of *Ca*. T. autotrophicus lacks nitrous oxide reductase (NosZ), the enzyme required to complete the final step of denitrification, in which N₂O is reduced to dinitrogen gas (31). This is consistent with the previous reports of an incomplete denitrification pathway in *Thioglobus,* a genus within the *Thioglobaceae* (27, 32). As discussed above, OMZs account for a large fraction of marine N₂O emissions (10–12), which primarily results from incomplete denitrification (11), and the high abundance of *Thioglobaceae* in OMZs (24), coupled with laboratory experiments by Shah et al. (31), suggests that chemoautotrophic members of the *Thioglobaceae* may be an important source of N₂O in OMZs.

Eastern boundary upwelling ecosystems (EBUEs) are among the most productive regions of the ocean, contributing up to 11% of global primary production (33, 34). Among EBUEs, the nBUS has been reported as the most productive, accounting for up to 40% of global EBUE production (35, 36), yet it is one of the most understudied OMZs in the world. The nBUS EBUE has high productivity due, in part, to year-round upwelling (34, 35) facilitated by trade winds that displace surface waters and transport cold, nutrient-rich water to the surface (37). The nBUS ecosystem is further influenced by regional circulation of water masses with distinct oxygen concentrations. Thus, large-scale and regional circulation patterns determine the composition of the upper central water layer and subsequently the oxygen balance over the Namibian shelf (38).

Although the nBUS is the most productive EBUE and is the source of ~13% of N₂O from EBUE OMZ environments (34, 39, 40), it remains relatively understudied compared to other EBUEs. Uncertainties in N₂O microbial production mechanisms in the nBUS point to a significant knowledge gap that creates uncertainty in modeling future global climate scenarios (41, 42). In particular, the potential role of abundant *Thioglobaceae* in incomplete denitrification and subsequent N₂O production across oxygen gradients has not been fully resolved in OMZ systems. To address this knowledge gap, we analyzed the microbial communities in the nBUS OMZ using 16S rRNA gene sequencing and paired these data with genome annotation and comparative genomic analysis of *Thioglobaceae*. Specifically, amplicon sequence variants (ASVs) were evaluated to assess the shifts in microbial diversity and community composition across oxygen gradients. The increase in relative abundance of *Thioglobaceae* as oxygen declined in the nBUS, and their dominance in other OMZs, as discussed above, led to genome analyses of this clade. The genomic data set included cultured representatives with complete genomes, as well as genomes from metagenome assemblies and single amplified genomes that were assembled to a minimum of 50% complete with less than 10% contamination (216 genomes total; 78% average genome completeness). These genomes represent microbes from the nBUS and the global ocean and were evaluated to resolve the metabolic potential of *Thioglobaceae* in key biogeochemical processes, including linked carbon, sulfur, and nitrogen cycles, and more specifically their potential to produce the potent greenhouse gas N₂O as a result of an incomplete denitrification pathway within OMZs.

## RESULTS

### Samples

In total, 46 samples that spanned depth and oxygen gradients were collected from 11 stations in the nBUS ecosystem off the coast of Namibia in April 2017 (Table 1). Samples were obtained from the surface to 972 m deep (Table 1). Water column temperatures ranged from 4°C to over 17°C, and salinity was an average of 35 psu (Table 1). DO concentrations were grouped into three categories: oxic (>2.0 mL/L), dysoxic (0.2–2.0 mL/L), and suboxic (0.0–0.2 mL/L) (43).

**TABLE 1:** Metadata and environmental data for 11 stations sampled in the nBUS

Station	Sample	Date	Time	Latitude	Longitude	DO (mL/L)	Salinity (psu)	Temperature (°C)	Depth (m)	DO category
1	18002	4/23/17	10:40	−18	11.78	2.1	35.48	15.43	1.7	Oxic
1	18002	4/23/17	10:40	−18	11.78	1.6	35.48	15.44	10.6	Dysoxic
1	18002	4/23/17	10:40	−18	11.78	1.4	35.48	15.11	20.65	Dysoxic
1	18002	4/23/17	10:40	−18	11.78	1.4	35.47	15.09	31.08	Dysoxic
2	18010	4/23/17	6:07	−18	11.63	3.54	35.6	16.8	20.7	Oxic
2	18010	4/23/17	6:07	−18	11.63	3.4	35.6	17.06	2	Oxic
2	18010	4/23/17	6:07	−18	11.63	1.53	35.55	15.62	81.45	Dysoxic
2	18010	4/23/17	6:07	−18	11.63	0.41	35.38	13.95	127.16	Dysoxic
3	18030	4/22/17	22:47	−18	11.28	6.25	35.62	17.86	3	Oxic
3	18030	4/22/17	22:47	−18	11.28	5.6	35.65	17.38	30	Oxic
3	18030	4/22/17	22:47	−18	11.28	5.42	35.63	17.73	10	Oxic
3	18030	4/22/17	22:47	−18	11.28	5.23	35.64	17.41	20	Oxic
3	18030	4/22/17	22:47	−18	11.28	3.3	34.55	4.01	971.6	Oxic
3	18030	4/22/17	22:47	−18	11.28	0.71	35.15	12.12	201	Dysoxic
3	18030	4/22/17	22:47	−18	11.28	0.68	34.54	6.08	600.97	Dysoxic
4	20010	4/21/17	20:40	−20	12.86	3.54	35.47	16.04	30.4	Oxic
4	20010	4/21/17	20:40	−20	12.86	3.1	35.47	16.08	20	Oxic
4	20010	4/21/17	20:40	−20	12.86	1.8	35.48	15.61	60.5	Dysoxic
4	20010	4/21/17	20:40	−20	12.86	0.08	35.43	14.31	90	Suboxic
5	20020	4/21/17	17:00	−20	12.68	3.5	35.48	16.78	10.89	Oxic
5	20020	4/21/17	17:00	−20	12.68	2.4	35.51	16.08	31.13	Oxic
5	20020	4/21/17	17:00	−20	12.68	0.17	35.38	13.91	118.1	Suboxic
5	20020	4/21/17	17:00	−20	12.68	0.02	35.5	14.93	80.8	Suboxic
6	20040	4/22/17	3:50	−20	12.33	2.64	35.5	16.55	20.27	Oxic
6	20040	4/22/17	3:50	−20	12.33	0.57	35.49	15.24	51.5	Dysoxic
6	20040	4/22/17	3:50	−20	12.33	0.42	35.41	14.15	101.17	Dysoxic
6	20040	4/22/17	3:50	−20	12.33	0.14	35.18	12.26	210	Suboxic
7	23002	4/19/17	17:56	−23	14.37	2.74	35.29	14.47	11	Oxic
7	23002	4/19/17	17:56	−23	14.37	1.1	35.29	13.95	21.1	Dysoxic
7	23002	4/19/17	17:56	−23	14.37	0	35.32	13.76	36.2	Suboxic
8	23020	4/19/17	23:00	−23	14.04	5.1	35.31	15.24	10.86	Oxic
8	23020	4/19/17	23:00	−23	14.04	1.4	35.27	13.83	30.3	Dysoxic
8	23020	4/19/17	23:00	−23	14.04	0.35	35.25	13.05	82	Dysoxic
8	23020	4/19/17	23:00	−23	14.04	0.16	35.23	12.83	120	Suboxic
9	23070	4/20/17	9:00	−23	13.14	5.4	35.26	16.36	21.5	Oxic
9	23070	4/20/17	9:00	−23	13.14	3.6	35.22	14.85	60.4	Oxic
9	23070	4/20/17	9:00	−23	13.14	1.12	34.82	9.46	306	Dysoxic
9	23070	4/20/17	9:00	−23	13.14	0.95	35.13	12.22	148.8	Dysoxic
10	GeoChem1-2	4/20/17	22:00	−22.2	14.22	0.99	35.44	14.85	2	Dysoxic
10	GeoChem1-2	4/20/17	22:00	−22.2	14.22	0.44	35.4	14.95	4.5	Dysoxic
10	GeoChem1-2	4/20/17	22:00	−22.2	14.22	0.07	35.43	14.55	11.5	Suboxic
10	GeoChem1-2	4/20/17	22:00	−22.2	14.22	0.02	35.43	14.38	21	Suboxic
11	GeoChem1-6	4/24/17	0:16	−18.4	11.3	2.6	35.53	16.16	21	Oxic
11	GeoChem1-6	4/24/17	0:16	−18.4	11.3	0.18	35.52	15.19	58.44	Suboxic
11	GeoChem1-6	4/24/17	0:16	−18.4	11.3	0.11	35.39	14	98.66	Suboxic
11	GeoChem1-6	4/24/17	0:16	−18.4	11.3	0.08	35.54	15.51	45	Suboxic

### Microbial community diversity

**Fig 1:**
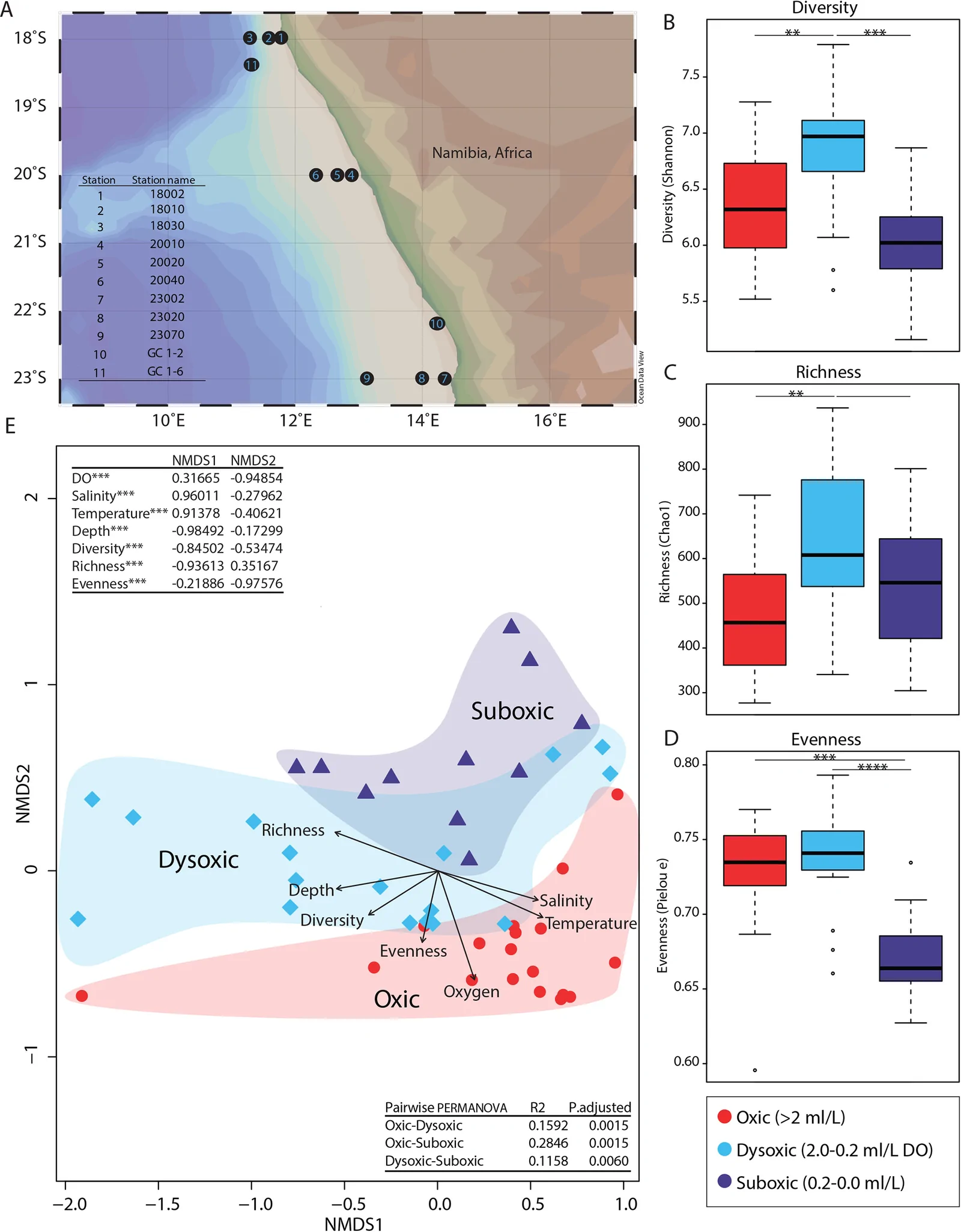
(**A**) Sampling stations off the coast of Namibia in the northern Benguela Upwelling System (nBUS). (**B–D**) Boxplots show diversity metrics of 16S rRNA gene sequence data. Microbial diversity, richness, and evenness were compared across oxic, dysoxic, and suboxic conditions. (**E**) Non-metric multidimensional scaling (NMDS) ordination of 16S rRNA gene sequence data. Environmental data were overlain on the ordination, with correlations between variables and ordination axes shown in the table in the figure. Corrected *P*-values are denoted by ** (*P* ≤ 0.01), *** (*P* ≤ 0.001), and **** (*P* ≤ 0.0001).

Analysis of the amplicon sequence variant (ASV) data from nBUS stations (Fig. 1A) revealed that microbial community diversity varied across oxic, dysoxic, and suboxic conditions. Specifically, diversity (Shannon diversity) was significantly higher in dysoxic samples compared to oxic and suboxic samples (Fig. 1B). Similarly, species richness (Chao1) was highest in dysoxic samples and significantly lower in oxic samples (Fig. 1C). Community evenness (Pielou’s evenness) was highest in the oxic and dysoxic samples and significantly lower in the suboxic samples (Fig. 1D).

### Microbial community structure

Normalized ASV data were evaluated using a non-metric multidimensional scaling (NMDS) ordination to assess community structure (Fig. 1E). Environmental data and diversity metrics were overlain onto the ordination, revealing that DO was an important factor structuring microbial communities, with samples from similar DO concentrations and depths clustering together (Fig. 1E). Sample clusters based on DO concentrations were significantly different, as determined using pairwise PERMANOVA. In addition to DO, depth, temperature, and salinity were also significantly correlated with NMDS ordination axes (Fig. 1E). The ordination-based patterns were consistent with the variation in diversity, richness, and evenness discussed previously (Fig. 1B through D). For example, diversity, richness, and evenness were highest in dysoxic samples, while diversity and evenness were lowest in suboxic samples and richness was lowest in oxic samples (Fig. 1B through E).

**Fig 2:**
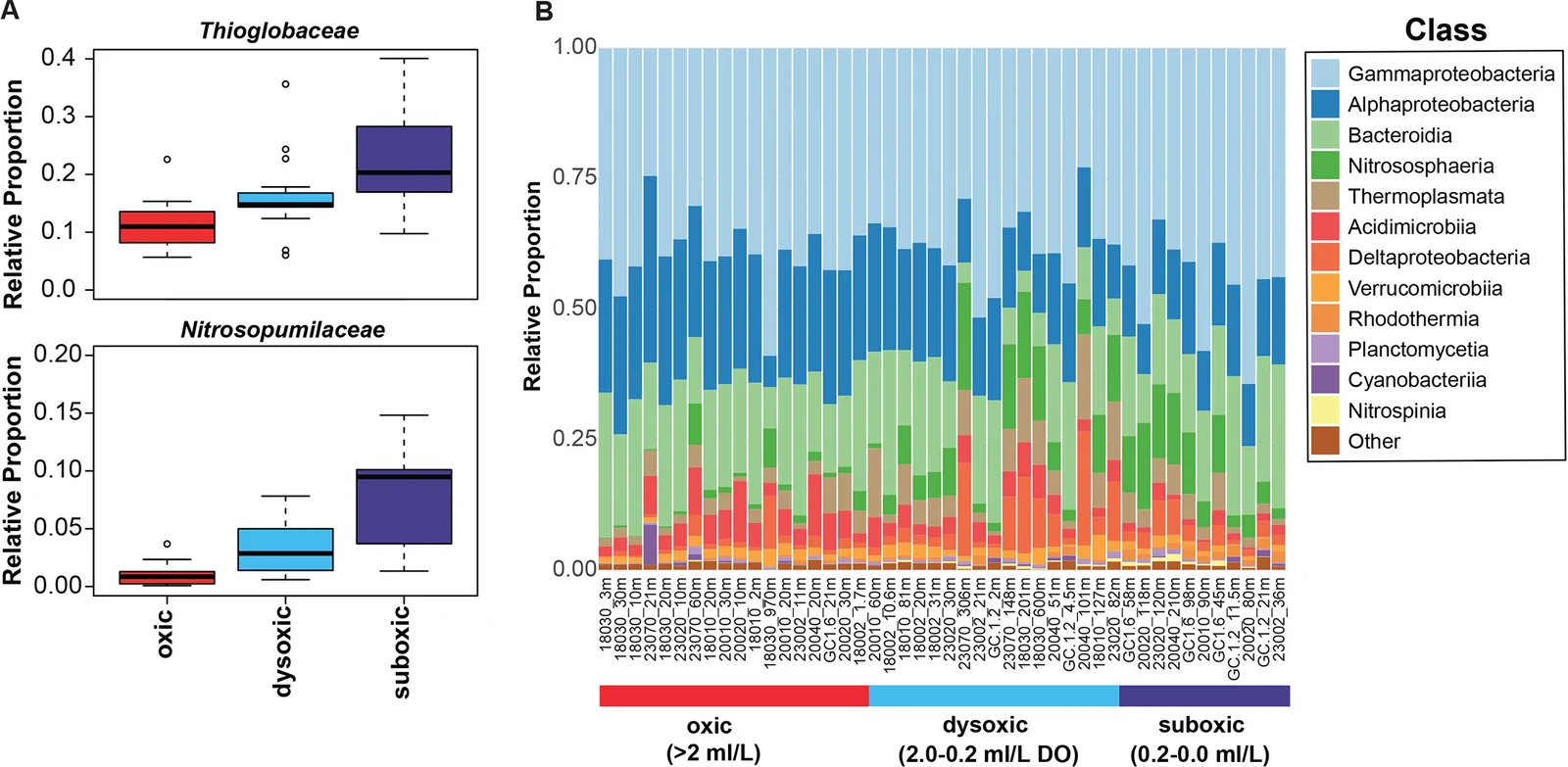
(**A**) The boxplots show the relative proportion of *Thioglobaceae* (Class *Gammaproteobacteria*) and *Nitrosopumilaceae* (Class *Nitrososphaeria*) from 16S rRNA gene sequence data in relation to oxygen concentrations. Boxes represent the interquartile range (IQR), center lines indicate medians, and whiskers represent 1.5 × IQR. (**B**) A bar graph of 16S rRNA gene data showing relative proportions of bacterial and archaeal classes. Only the most abundant groups are shown, with less abundant classes grouped as “Other.” Samples are sorted by DO concentration. In both panels **A** and **B**, color coding is as follows: suboxic (purple), dysoxic (light blue), and oxic (red).

Of the 5,463 total ASVs, *Thioglobaceae* ASVs comprised up to 40% of the relative abundance (minimum of 6%; Fig. 2A) and were observed in all samples, making *Thioglobaceae* one of the most abundant microbial groups in the nBUS. A linear regression revealed a significant inverse correlation between *Thioglobaceae* relative abundance and DO (β = −0.023 ± 0.005, *P* = 1.1 × 10⁻⁴). SIMPER analysis was used to identify ASVs contributing to differences in community composition among oxygen categories, with pairwise comparisons conducted for oxic-dysoxic, oxic-suboxic, and dysoxic-suboxic samples. This analysis revealed that 138 ASVs, including *Thioglobaceae* ASVs, contributed significantly to differences among categorical oxygen clusters in the NMDS ordination. The normalized abundances of these individual ASVs differed significantly among oxygen categories based on Kruskal–Wallis tests with false discovery rate correction (FDR; *P* < 0.05). These ASVs were predominantly affiliated with the classes *Gammaproteobacteria*, *Thermoplasmata*, and *Nitrososphaeria*, consistent with the class-level summary shown in Fig. 2B. In addition, the summed normalized abundance of the 138 SIMPER-associated ASVs differed significantly among oxygen categories (Kruskal–Wallis test, H = 8.37, df = 2, *P* = 0.015). Of these ASVs, one *Thioglobaceae* ASV was the strongest contributor to community differences in the oxic-suboxic and oxic-dysoxic comparisons.

Across the full data set, 134 classes were identified, with *Gammaproteobacteria*, *Alphaproteobacteria*, *Bacteroidia*, *Nitrososphaeria*, and *Thermoplasmata* dominating the nBUS communities at the class level (Fig. 2B). Within these broader classes, family-level patterns varied across the DO gradient. For example, *Thioglobaceae* decreased in relative abundance with increasing DO concentrations (Fig. 2A). *Nitrosopumilaceae* in the *Nitrososphaeria* (Fig. 2A) showed a similar pattern and was significantly negatively correlated with DO concentrations (β = −1,487.6 ± 279.4, *P* = 1.6 × 10⁻⁶). In contrast, some classes were nearly invariant across the DO gradient, such as *Poseidoniales* (data not shown) in the *Thermoplasmata* (Fig. 2B).

### Genome information, classification, and average nucleotide identity

The 216 medium-to-high quality genomes discussed herein were derived from microbes sampled in the global ocean, including the nBUS (Table S1). The data set includes the first cultured *Thioglobaceae* (previously referred to as SUP05) representative, *Ca*. T. autotrophicus strain EF1 (44), as well as the more recent isolates EF2 and EF3 from Effingham Inlet, British Columbia (45). The data set also includes the genome of *Ca*. T. sp. strain NP1, from the North Pacific subpolar gyre (46). Additional genomes were obtained from cultured and uncultured microbes sampled in the Peru upwelling region OMZ, such as *Ca*. T. perditus (47), and from numerous *Thioglobaceae* (SUP05) sampled from the OMZ off the Chilean coast and in the seasonally anoxic Saanich Inlet in British Columbia (27).

*Thioglobaceae* genomes were classified as belonging to the genera *Thioglobus*, SUP05, *Pseudothioglobus*, SZUA-1055, *Thiodubiliella*, DUCF01, and JAAOIF01 (Fig. S1). To link ASV and genomic data sets, we compared 16S rRNA genes using BLAST to assess sequence similarity between *Thioglobaceae* ASVs and genomes. Of the 97 genomes with 16S rRNA genes recovered in the assembly (Table S1), 91 had BLAST hits to ASVs, with an average of 100% similarity; all but one match showed ≥98% identity. When ASV data were converted to relative abundances, *Thioglobaceae* ASVs represented in the genomes accounted for an average of 98% (range: 89%–100%) of the total *Thioglobaceae* relative abundance in the ASV data set.

The *Thioglobaceae* genomes analyzed had an average genome completeness of 78% and an average contamination of 1.5%. Average nucleotide identity (ANI) analysis of these genomes revealed ANI values ranging from >75% to 99% in similarity within genera and in some instances across the *Thioglobus*, SUP05, SZUA-1055, and *Pseudothioglobus* genera (Fig. S1). Generally, ANI values were <75% when comparing genomes across the different taxonomic groups (Fig. S1).

### Carbon fixation and utilization

**Fig 3:**
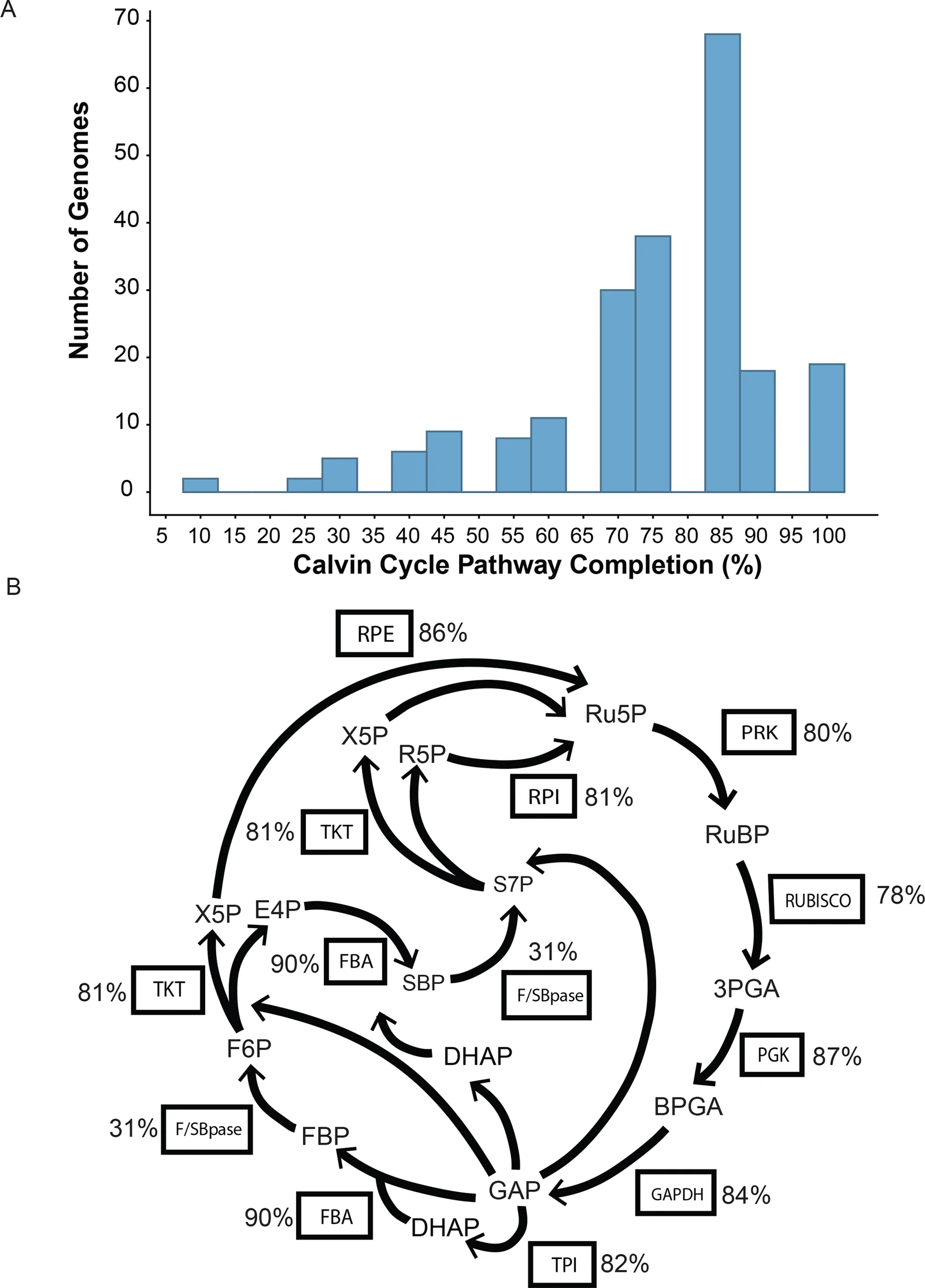
Carbon fixation pathway via the canonical Calvin-Benson cycle. (**A**) Histogram showing the distribution of pathway completeness across analyzed genomes. (**B**) Enzymes in the canonical Calvin-Benson cycle are shown in boxes as ribulose-1,5-bisphosphate carboxylase/oxygenase (RuBisCO), phosphoglycerate kinase (PGK), glyceraldehyde-3-phosphate dehydrogenase (GAPDH), triosephosphate isomerase (TPI), fructose-bisphosphate aldolase (FBA), fructose-1,6-bisphosphatase/sedoheptulose-1,7-bisphosphatase (F/SBpase), transketolase (TKT), ribose 5-phosphate isomerase (RPI), ribulose-phosphate 3-epimerase (RPE), and phosphoribulokinase (PRK). The percentages next to the enzyme represent the percentage of genomes coding for each enzyme. The products of each step of the pathway are shown in black between the arrows.

Analysis of carbon fixation pathways in *Thioglobaceae* revealed that all genomes encoded one or more genes in the Calvin-Benson (CB) cycle. Of these, 9% (19 of 216) encoded a complete carbon fixation pathway via the canonical CB cycle (Fig. 3), as defined in the study by Hudson et al. (48). SUP05 and *Thioglobus* genera were the only groups whose members were able to carry out complete carbon fixation via the canonical CB cycle as they encoded all genes in this pathway including the bifunctional enzyme fructose-1,6-bisphosphatase/sedoheptulose-1,7-bisphosphatase (F/SBpase; Fig. 3B). This enzyme hydrolyzes fructose-1,6-bisphosphate and sedoheptulose-1,7-bisphosphate and was encoded in 31% of the genomes (68 of 216). Twenty-eight percent (60 of 216) of the genomes lacked only F/SBpase while encoding the remainder of the canonical CB cycle. These genomes spanned multiple genera, including SUP05, *Thioglobus*, *Pseudothioglobus*, SZUA-1055, *Thiodubiliella*, DUCF01, and JAAOIF01. This group includes the *Ca*. T. autotrophicus strain EF1 and cultured EF2 and EF3 isolates with complete genomes (44, 45).

Carbon utilization was also analyzed in all genomes, revealing that the tricarboxylic acid cycle (TCA) was encoded by all genomes but with varying degrees of pathway completion. For example, key components of the TCA cycle, such as 2-oxoglutarate dehydrogenase (K00164), were lacking in 69% (149 of 216) of the genomes, including most *Thioglobus* and all SUP05. This suggested that the majority of *Thioglobaceae*, particularly *Thioglobus* and SUP05, carry out a modified TCA cycle. In contrast, nearly all *Pseudothioglobus* encoded this enzyme. In 60 of the genomes that lacked 2-oxoglutarate dehydrogenase genes, malate dehydrogenase (K00024), another key component of the TCA cycle, was also missing. Genomes lacking both genes were predominantly classified as *Thioglobus*; however, all other *Thioglobaceae* genera were also represented in this group.

### Aerobic respiration

Sixty-nine percent (150 of 216) of the genomes encoded cytochrome c oxidase subunits I–III (CoxABC), a low-oxygen-affinity enzyme. Nearly 59% (127 of 216) of the genomes analyzed encoded cbb3-type cytochrome c oxidase subunits I–II, a high-oxygen-affinity enzyme. All *Thioglobaceae* clades had representatives encoding this enzyme. Both low- and high-oxygen-affinity enzymes were encoded in 94 genomes. In total, only 15% (33 of 216) of the genomes lacked both low- and high-oxygen-affinity enzymes.

### Denitrification

**Fig 4:**
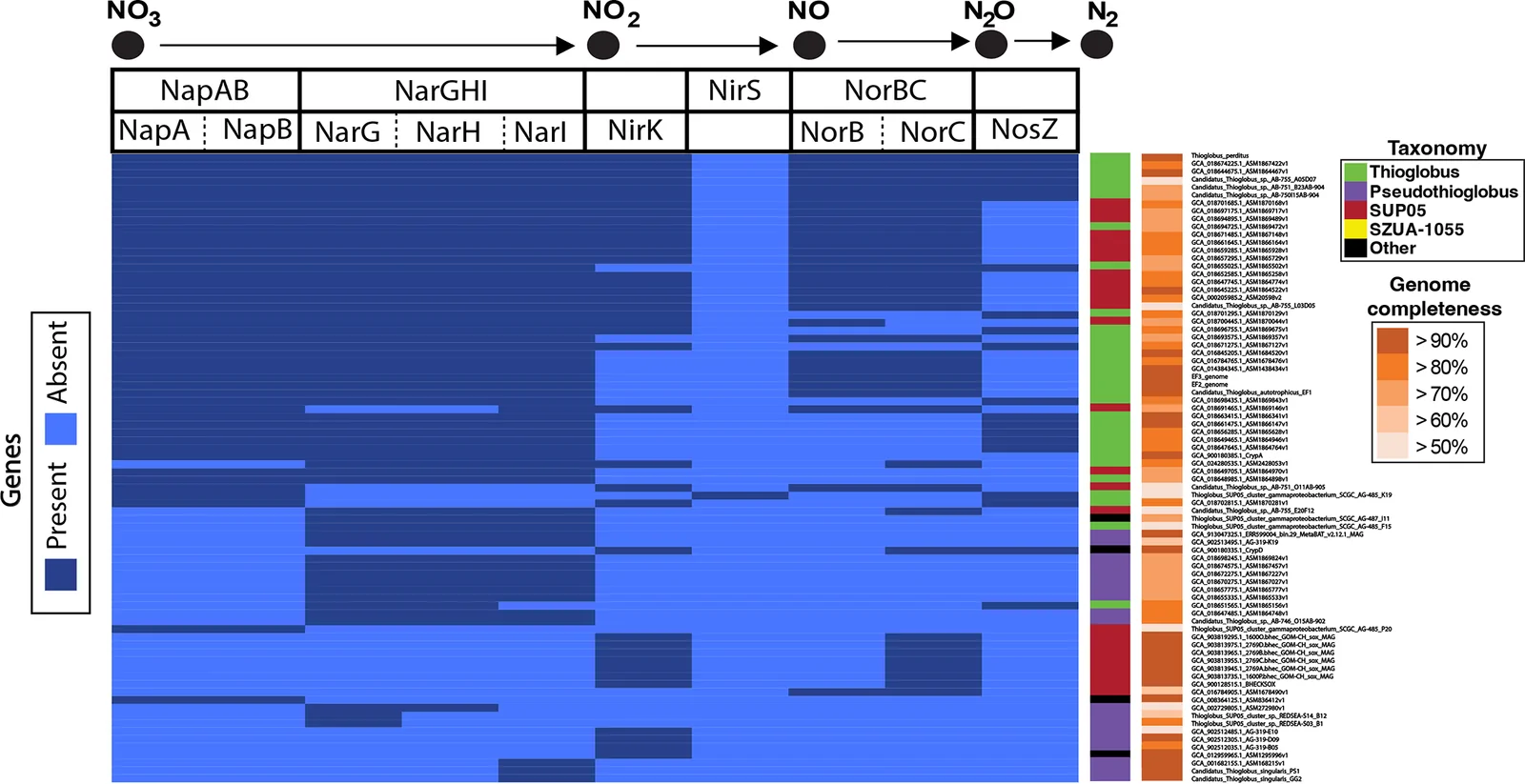
Dissimilatory denitrification pathway with only those genomes that encoded at least one step in this pathway shown here. The presence or absence of genes involved in dissimilatory denitrification is represented by dark blue (present) and light blue (absent), respectively. Genome completeness is color-coded based on percent completeness values. Further, the genomes are color-coded by taxonomic classification at the genus level. In the legend, black indicates the “other” category and shows clades represented by less than three genomes. The enzymes of each pathway are labeled at the top of the heatmap, along with a depiction of the pathway where black circles represent the products and/or intermediates. The enzymes with multiple subunits are divided by dashed lines. The enzymes are abbreviated as follows: nitrate reductase subunit A (NapA), nitrate reductase subunit B (NapB), respiratory nitrate reductase subunit G (NarG), respiratory nitrate reductase subunit H (NarH), respiratory nitrate reductase subunit I (NarI), nitrite reductase subunit K (NirK), nitrite reductase subunit S (NirS), nitric oxide reductase subunit B (NorB), nitric oxide reductase subunit C (NorC), and nitrous oxide reductase (NosZ).

In addition to aerobic respiration, 37% (80 of 216) of the genomes, from both cultured and uncultured representatives, encoded genes associated with denitrification, with varying degrees of pathway completion. This included *Thioglobus*, *Pseudothioglobus*, and SUP05, with fewer from DUCF01, *Thiodubiliella*, and SZUA-1055. Of the 80 genomes, only six *Thioglobus*, including *Ca*. T. perditus (47), encoded all the necessary genes for complete denitrification, including nitrate reductase (*napAB*), respiratory nitrate reductase (*narGHI*), nitrite reductase subunit K (*nirK*) (only one genome encoded *nirS*), nitric oxide reductase (*norBC*), and nitrous oxide reductase (*nosZ)* (Fig. 4). The final step of denitrification is the conversion of nitrous oxide (N₂O) to dinitrogen gas (N₂), which is mediated by *nosZ* (Fig. 4). This gene was missing in 14 genomes that encoded all other enzymes in the denitrification pathway (Fig. 4). These genomes included 13 classified as SUP05 and one classified as *Thioglobus*. The remaining 60 genomes encoded some steps of the denitrification pathway but not a complete denitrification pathway (Fig. 4).

### Sulfur oxidation

**Fig 5:**
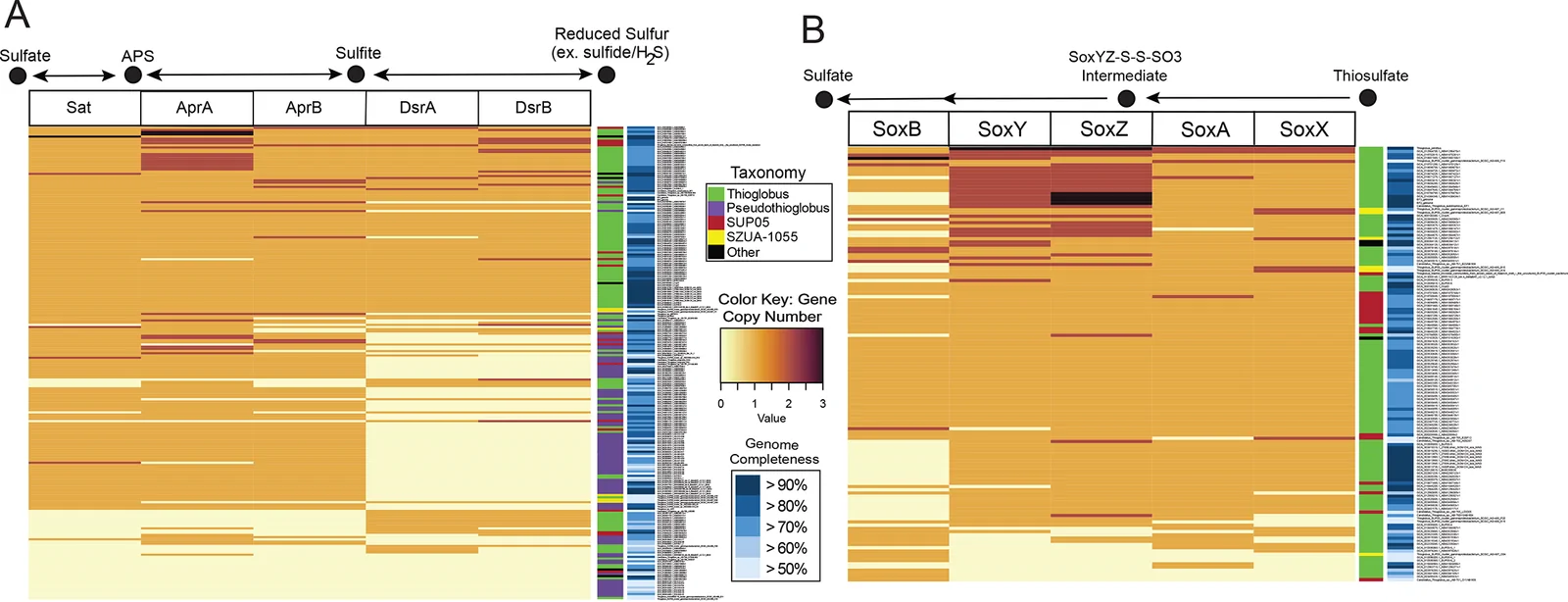
(**A**) Sulfur oxidation via the reverse dissimilatory sulfate reduction (rDSR) pathway and (**B**) sulfur oxidation via the Sox enzyme complex with gene copy number. The number of gene copies per genome is indicated by the color key. Genomes are color-coded by taxonomic classification at the genus level. Black, indicating the “other” category, represents clades with fewer than three genomes. Genome completeness is color-coded based on percent completeness. The Sox enzyme complex is abbreviated as follows: sulfate thiohydrolase (SoxB), sulfur-oxidizing protein subunit Y (SoxY), sulfur-oxidizing protein subunit Z (SoxZ), L-cysteine S-thiosulfotransferase subunit A (SoxA), and L-cysteine S-thiosulfotransferase subunit X (SoxX). The enzymes for sulfur oxidation via the rDSR pathway are abbreviated as follows: sulfate adenylyltransferase (Sat), adenylylsulfate reductase, subunit A (AprA), adenylylsulfate reductase, subunit B (AprB), dissimilatory sulfite reductase alpha subunit (DsrA), and dissimilatory sulfite reductase beta subunit (DsrB). Black circles represent products and/or intermediates. Double arrows indicate that the steps in the pathway can proceed in either direction.

Genome annotation revealed that 91% (196 of 216) of the genomes encoded genes associated with sulfur oxidation via the reverse dissimilatory sulfate reduction (rDSR) pathway (Fig. 5A). Of the 196 genomes, 82 encoded the complete pathway, which included the genes sulfate adenylyltransferase (*sat*), adenylylsulfate reductase, subunit A (*aprA*), adenylylsulfate reductase, subunit B (*aprB*), dissimilatory sulfite reductase alpha subunit (*dsrA*), and dissimilatory sulfite reductase beta subunit (*dsrB*) (Fig. 5A). The 82 genomes encoding complete pathways included 68 *Thioglobus*, eight SUP05, three *Thiodubiliella*, two SZUA-1055, and one JAAOIF01. In contrast, 114 of the 196 genomes encoded incomplete pathways, including 67 *Pseudothioglobus*, 33 *Thioglobus*, 10 SUP05, and 4 SZUA-1055 (Fig. 5A).

Genes involved in the Sox multi-enzyme complex (SoxABXYZ) are representative metabolic markers for inorganic sulfur oxidation via the oxidation of thiosulfate to sulfate (49, 50). Gene annotations revealed that 63% (135 of 216) of the *Thioglobaceae* genomes encoded some *sox* genes for sulfur oxidation, including members of the genera SUP05, *Thioglobus*, SZUA-1055, *Thiodubiliella*, and JAAOIF01 (Fig. 5B). Of the 135 genomes with *sox* genes, 73 encoded all genes required for sulfur oxidation (Fig. 5B). This included 54 *Thioglobus*, 13 SUP05, three SZUA-1055, two *Thiodubiliella*, and one JAAOIF01 that encoded the full Sox multi-enzyme complex (Fig. 5B).

## DISCUSSION

### Dissolved oxygen and microbial community structure

As global ocean temperatures rise, DO levels are expected to decline, fueling the expansion of global OMZs (5, 14). In OMZs, numerous studies have demonstrated that low DO strongly influences microbial community dynamics (2). Here, we investigated changes in diversity and community composition along a DO gradient in the nBUS and identified DO as an important factor shaping microbial community diversity and structure. For example, community diversity was significantly lower in suboxic samples as compared to dysoxic samples, which can largely be attributed to significantly lower evenness in suboxic samples. Our findings are consistent with reports in which microbial diversity metrics were determined in OMZs. Specifically, community diversity has been reported to decrease within low-DO environments in OMZs (17, 18). In our samples, community richness was highest in dysoxic samples and significantly lower in both oxic and suboxic samples. This observation aligns with the study by Beman and Carolan (17), who reported a unimodal pattern between DO and bacterial richness, with a peak in richness at the edges of the OMZ and decreasing richness in the interior. This is also consistent with an OMZ off British Columbia, where a large decline in richness was observed when transitioning into a hypoxic environment (20), underscoring the relationship between microbial richness and DO (16).

The significant decrease in community evenness in suboxic samples compared to oxic and dysoxic samples suggested that shifts in DO are associated with changes in the distribution of specific microbial groups. Previous reports revealed the dominance of distinct microbial groups in low-DO samples (15–17), which likely influences community evenness. Similarly, we found that the relative abundance of *Thioglobaceae* (and *Nitrosopumilaceae*) increased significantly as DO decreased. In fact, *Thioglobaceae* comprised up to 40% of the microbial community in the low-DO samples, consistent with other OMZ studies (27, 28, 47, 51), including the nBUS (24, 26, 52).

### Genomic analysis of the metabolic function of Thioglobaceae

#### Carbon

The important role of *Thioglobaceae* in the marine carbon cycle, with both carbon fixation and heterotrophy reported for this clade, has been extensively reviewed (51). In our analysis, only SUP05 and *Thioglobus* encoded a complete carbon fixation pathway through the canonical Calvin-Benson (CB) cycle. The canonical CB cycle and the noncanonical CB cycle are set apart by fructose-1,6-bisphosphate and sedoheptulose-1,7-bisphosphate hydrolysis activity, which is mediated by the bifunctional enzyme F/SBpase (48). The canonical CB cycle includes F/SBpase, which was observed in less than a third of the genomes. Most of the remaining genomes lacked F/SBpase, suggesting that the majority of *Thioglobaceae* have the genetic potential to carry out carbon fixation through an alternative pathway of the canonical CB cycle as all other components of the canonical CB cycle were present. Genomes lacking F/SBpase included all three *Ca*. T. autotrophicus isolates, EF1, EF2, and EF3, all of which have complete genome sequences (44, 45). This suggested that the absence of the F/SBPase gene in these genomes is real and not attributable to incomplete genome assembly. A possible alternative pathway for sulfur-oxidizing chemoautotrophs is the replacement of F/SBpase with inorganic pyrophosphate-dependent phosphofructokinases (PPi-PFKs), which operate in reverse (53).

Notably, our analysis revealed that at least one representative of each genus within the *Thioglobaceae* encoded complete carbon fixation through either the canonical CB or noncanonical CB pathway. Recently, *Ca*. T. autotrophicus isolates EF2 and EF3 were shown to have the genetic potential to fix inorganic carbon consistent with a noncanonical CB pathway (45), which was originally reported in strain EF1 (44) and confirmed experimentally by Shah et al. (29). This finding is important given that the SUP05 clade (as it is referred to in the literature) has been reported to use the energy obtained from oxidizing reduced sulfur for autotrophic carbon fixation with ribulose-1,5-bisphosphate carboxylase/oxygenase (RuBisCO) (27, 51, 54), which was identified in 78% of the genomes analyzed, including strains EF1, EF2, and EF3. Overall, the ability to fix carbon either via the canonical CB or noncanonical CB pathway was encoded in a large number of the analyzed genomes and was observed across all genera. These genomes spanned taxonomic groups and represented microbes sampled from the global ocean, suggesting that *Thioglobaceae* as a whole are important contributors to the marine carbon cycle through carbon fixation.

Analysis of carbon utilization by *Thioglobaceae* also revealed that key components of the TCA cycle, such as 2-oxoglutarate dehydrogenase, were lacking in many genomes. This suggested that *Thioglobaceae* may utilize a “horseshoe TCA cycle,” an incomplete TCA pathway that lacks the oxidative, carbon dioxide-producing steps, thus retaining carbon for biosynthesis (51, 55). In contrast, the canonical TCA cycle fully oxidizes carbon intermediates to carbon dioxide, maximizing energy yield but reducing carbon available for biosynthesis. The horseshoe TCA cycle is particularly relevant in the context of the nBUS as it may allow *Thioglobaceae* to conserve fixed carbon and prioritize biosynthetic processes in OMZs, where DO is limited and alternative electron acceptors such as nitrate yield less energy (51, 55).

#### Aerobic and anaerobic respiration

Both low- and high-affinity oxygen enzymes, cytochrome c oxidase subunits I–III (CoxABC) and cbb3-type cytochrome c oxidase subunits I–II, were identified in nearly half of the analyzed genomes, indicating the capacity for aerobic respiration. The presence of both low- and high-affinity enzymes in these genomes suggested a potential adaptive mechanism for varying oxygen conditions, which has been examined in laboratory experiments with *Ca*. T. autotrophicus strain EF1 (44). For example, Shah et al. (31) showed that EF1 had the same growth rates and reached the same cell densities when grown under oxygen-saturated conditions or when oxygen was absent. Similarly, Mattes et al. (30) grew EF1 under high- and low-DO conditions and reported increased gene expression and carbon fixation under oxic conditions. The increase in carbon fixation under oxic conditions as reported in Mattes et al. (30) is ecologically relevant as oxygen availability can influence carbon fixation rates in *Thioglobaceae*.

In addition to aerobic respiration, over a third of the genomes analyzed encoded at least one step in dissimilatory denitrification, with only six *Thioglobus* genomes encoding complete denitrification. Further, several genomes lacked genes coding for NosZ, the enzyme that mediates the final denitrification step. This denitrification pathway was truncated following nitric oxide reductase (NorBC), which converts NO to N₂O. These new findings, coupled with experiments with *Ca*. T. autotrophicus strain EF1 showing N₂O production (31), indicated that incomplete denitrification is common within *Thioglobaceae*. Thus, the capacity for complete denitrification (via NosZ) is limited, suggesting widespread potential for N₂O accumulation. In particular, the nBUS has elevated sea-air fluxes of N₂O (34), with Frame et al. (40) reporting fluxes as high as 46 µmol m⁻² d⁻¹. Dangl et al. (52) presented a *Thioglobaceae* MAG, assembled from an nBUS sample, which lacked nosZ, and suggested that it may be an important incomplete denitrifier potentially involved in N₂O production in this ecosystem. The inclusion of *Thioglobaceae* genomes from the global ocean in our study expands our findings to other OMZs. Placing *Thioglobaceae* in a global OMZ context helps reconcile biogeochemical modeling results that showed an order-of-magnitude increase in N₂O cycling rates in the suboxic Eastern Tropical North Pacific when incomplete denitrification pathways, such as those encoded by the abundant Thioglobaceae, were incorporated (56).

#### Sulfur oxidation

We identified several SUP05 and *Thioglobus* genomes containing key enzymes in known sulfur oxidation pathways that have the potential to oxidize a range of reduced sulfur compounds. Specifically, *Thioglobus,* SUP05, and a small number of other *Thioglobaceae* genomes encoded essential *sox* genes, which would enable sulfur oxidation via the Sox multi-enzyme complex. The presence of the Sox multi-enzyme complex would enable energy acquisition through the oxidation of reduced inorganic sulfur compounds, including thiosulfate and, depending on the composition of the Sox complex, sulfide (57). The *Thioglobaceae* (previously referred to as SUP05) display a high affinity for sulfide (58) and are recognized as key contributors to sulfide oxidation in OMZs (59).

In total, 40% of the genomes, predominantly classified as *Thioglobus,* encoded the complete pathway for sulfur oxidation via the reverse dissimilatory sulfate reduction (rDSR) pathway, including the key genes dissimilatory sulfite reductase (*dsrAB*), adenylylsulfate reductase (*aprAB*), and sulfate adenylyltransferase (*sat*). *Thioglobus* and SUP05 have the genetic potential to use the energy gained from oxidizing reduced inorganic sulfur to drive carbon fixation. Given that these clades are among the most abundant microbes in OMZs, their role in sulfur oxidation may be ecologically consequential. For instance, toxic hydrogen sulfide (H_2_S) is reported to accumulate in the nBUS (47). Massive fish kills off the African coast (60, 61), caused by an accumulation of H_2_S, may be mitigated by high abundances of *Thioglobaceae* (referred to as SUP05) in the nBUS and their detoxification of these sulfidic waters (26), which highlights the possible role of SUP05 and *Thioglobus* in mediating sulfur cycling and mitigating H_2_S toxicity in OMZs.

Collectively, our analyses revealed that DO influenced microbial community diversity and structure, with significant increases in the relative abundance of *Thioglobaceae* as DO concentrations declined in the nBUS. Comparing ASV and genomic data sets suggested that the abundant *Thioglobaceae* were captured in the nBUS and were represented in global ocean genomes analyzed here. Genomic analysis revealed that *Thioglobaceae* have the genetic capacity to be important mediators of carbon, nitrogen, and sulfur cycling in the marine environment. For example, *Thioglobus* and SUP05 genomes encoded the necessary genes to fix carbon via the canonical CB pathway, while other *Thioglobaceae* may do so by a noncanonical CB pathway. Further, these genomes encoded low- and high-affinity enzymes for aerobic respiration, as well as enzymes involved in denitrification. This suggested that *Thioglobaceae* are potentially metabolically active across an oxygen gradient, from oxic to suboxic, using different metabolic strategies for respiration, which, as discussed previously, has implications for carbon fixation rates that may decline in parallel with decreasing DO concentrations. Importantly, when oxygen concentrations are low and *Thioglobaceae* dominate, the documented absence of genetic machinery to convert N₂O to N₂ in several genomes indicated that many Thioglobaceae lack the genetic capacity to reduce N₂O to N₂, supporting their potential role in N₂O accumulation and flux from OMZs to the atmosphere. Finally, *Thioglobus* and SUP05 genomes encoded Sox and sulfur oxidation pathways, which were observed in other *Thioglobaceae* genomes but with varying degrees of pathway completion. Thus, declining DO concentrations in the global ocean could be met with increasing abundances of a microbial group that can detoxify the sulfidic water column yet exhibits declining carbon dioxide fixation rates, while simultaneously producing N₂O, a potent greenhouse gas, through incomplete denitrification.

## MATERIALS AND METHODS

### Study site and data collection

Seawater was collected from 11 stations off the coast of Namibia, Africa, in the nBUS on the R/V Mirabilis in April 2017 during austral fall (Table 1; Fig. 1A). A total of 46 samples were collected at various depths (ranging from 2 to 972 m) across an oxygen gradient (Table 1). Conductivity, temperature, and depth (CTD) and oxygen sensors were used to determine seawater properties. Oxygen concentrations were verified using the Winkler method (62). At each station, 5–10 L of seawater was collected at each depth. Seawater was pre-filtered using a 2.7-μm filter and concentrated on a 0.22-μm Millipore Sterivex filter. RNAlater was added to the Sterivex filter, which was stored at –80°C until DNA extraction.

### DNA extraction and 16S rRNA gene sequencing

DNA was extracted from one-half of the Sterivex filter, following the protocol described in Gillies et al. (63). Briefly, DNA was extracted using a CTAB extraction buffer (10% CTAB [hexadecyltrimethylammonium bromide], 1 M NaCl, and 0.5 M phosphate buffer, at pH 8) with 0.1 M ammonium aluminum sulfate and 25:24:1 phenol:chloroform:isoamyl alcohol paired with bead beating via FastPrep-24 (MP Biomedicals). Genomic DNA was purified with a QIAGEN AllPrep DNA/RNA Kit (Valencia, CA, USA). The purified DNA was quantified using a Qubit2.0 Fluorometer (Life Technologies, Grand Island, NY, USA).

The microbial community structure was determined by sequencing the V4 region of 16S rRNA genes in all 46 samples at Argonne National Laboratory’s Environmental Sample Preparation and Sequencing Facility (RRID:SCR_011062). Specifically, 16S rRNA genes were amplified from 10 ng of purified genomic DNA in duplicate using the modified archaeal and bacterial primers 515FB and 806RB (these primers target the V4 region; 64, 65) in accordance with the protocol described by Caporaso et al. (66, 67) and used by the Earth Microbiome Project (http://www.earthmicrobiome.org/emp-standard-protocols/16s/), with a slight modification: the annealing temperature was 60°C. Amplicons were sequenced using an Illumina MiSeq in the 250 × 250 bp mode. A total of 5.75 million sequences were generated for the 46 samples.

### Bioinformatics, taxonomy, and statistical analyses of 16S rRNA gene sequences

Raw sequences were demultiplexed using QIIME 2 (ver. 2018.6) (68). Demultiplexed reads were quality filtered, including chimera removal, and joined using DADA2 (69) with default parameters. The resulting ASV table was normalized using the Wrench package with the W2 estimator and defaults as recommended (70). ASV taxonomy was assigned with the Genome Taxonomy Database (GTDB; release 226) (71) in QIIME 2 using classify-sklearn. Alpha diversity metrics were calculated from ASV tables generated by multiple rarefactions, which were used to determine Shannon diversity (72), richness (Chao1) (73), and evenness (Pielou’s evenness) (74) using QIIME 2. To test for significant differences in alpha diversity metrics and normalized ASV abundances between samples with different DO concentrations, Kruskal-Wallis rank-sum tests with Benjamini-Hochberg correction were used.

The Wrench-normalized ASV table was analyzed using non-metric multidimensional scaling (NMDS) in R with the metaMDS command in the vegan package (75). Environmental variables and alpha diversity values were analyzed in relationship to NMDS axes with 999 permutations of the data. The simper function (similarity percentage, SIMPER) was used to evaluate the contribution of ASVs to Bray-Curtis dissimilarity, with oxic, dysoxic, and suboxic groupings. This was followed by a Dunn’s test, which is a nonparametric pairwise multiple comparisons statistic (76), to test for significant differences in abundance based on DO concentrations. The effect of DO concentrations on microbial community composition was tested using the distance matrix with permutational multivariate analysis of variance (PERMANOVA) with the *adonis2* command. The pairwiseAdonis package (77) was used to carry out multilevel pairwise comparisons, with *P*-values adjusted with an FDR correction. Beta-dispersion (betadisper, type = centroid, permutest with 999 permutations) was not significantly different among the treatments.

### Genome similarity, classification, and gene annotation

The Integrated Microbial Genomes Database (IMG) and GTDB were used to search for genomes historically classified as SUP05 and more generally for all genomes in the *Thioglobaceae* family. This search resulted in 324 publicly available genomes identified in and downloaded from IMG or identified in GTDB and downloaded from the National Center for Biotechnology Information (NCBI). CheckM (ver. 1.1.3) (78) was used to determine genome completeness and contamination. In total, 216 genomes that met the criteria of medium (>50% complete and <10% contamination) to high quality (>90% complete and <5% contamination; Table S1) were identified. These genomes represent both cultured and uncultured microbes. Taxonomy was assigned to each genome using the Genome Taxonomy Toolkit (GTDB-Tk, ver. 2.3.0 with the R08-RS214 database) with the gtdbtk classify_wf default settings (79). This classification uses up to 120 bacterial single-copy marker proteins. Genomes were annotated using DRAM (ver. 1.1.1) (80), which performs *de novo* gene prediction and functional annotation against the KEGG (81), UniRef90 (82), PFAM (83), dbCAN (84), and MEROPS (85) databases. In DRAM, Prokka (ver. 1.14.0) (86) was used to identify 16S rRNA genes. BLASTn (87) was used to compare 16S rRNA gene ASV data from the nBUS data set to those genes in the genomes analyzed here. Average nucleotide identity (ANI) was calculated using sourmash (88) with the following settings: sketch dna -p scaled=1000,k=21,k=31,k=51, and compare --containment --ani --ksize 31 to compare genetic relatedness among prokaryotic strains.

## Data Availability

All 16S rRNA sequences are available through NCBI (PRJNA1251143) and on the Mason lab server (http://mason.eoas.fsu.edu) in the nBUS directory. Publicly available genomes were downloaded from IMG and NCBI.

## SUPPLEMENTAL MATERIAL

Figure S1 msphere.00751-25-s0001.tif Genome count per genus and ANI for all 216 genomes.

Table S1 msphere.00751-25-s0002.xlsx Genomes analyzed and their associated metadata.

ASM does not own the copyrights to Supplemental Material that may be linked to, or accessed through, an article. The authors have granted ASM a non-exclusive, world-wide license to publish the Supplemental Material files. Please contact the corresponding author directly for reuse.
